# Human exposure to pesticides and thyroid cancer: a worldwide systematic review of the literatures

**DOI:** 10.1186/s13044-023-00153-9

**Published:** 2023-05-15

**Authors:** Fatemeh Norouzi, Ismaeil Alizadeh, Maryam Faraji

**Affiliations:** 1 Department of Environmental Health Engineering, Faculty of Public Health, Kerman University of Medical Sciences, Kerman, Iran; 2 Research Center of Tropical and Infectious Diseases, Kerman University of Medical Sciences, Kerman, Iran; 3 Environmental Health Engineering Research Center, Kerman University of Medical Sciences, Kerman, Iran

**Keywords:** Neoplasms, Pesticide exposure, Risk, Papillary thyroid carcinoma

## Abstract

Thyroid cancer is considered as one of the most prevalent cancers in the world. Some pesticides can play a role as a potentially important risk factor in thyroid cancer by affecting thyroid morphology and thyroid hormone homeostasis. The aim of present study was to systematically review the available epidemiological evidence for human exposure to pesticides and thyroid cancer. Articles were searched in PubMed, Scopus and Web of Science by suitable keywords from January 2000 to May 2021. Standard techniques for systematic reviews were followed in the current study and results reported according to Preferred Reporting Items for Systematic reviews and Meta-Analyses (PRISMA) guidelines. Based on the inclusion and exclusion criteria, finally seven studies including four cohort studies and three case-control studies were reviewed. Organochlorines (OCPs) in more cases, Organophosphates (OPs) and Carbamates insecticides, herbicides and fungicides were the studied pesticides. Inconsistent results were reported in the surveyed articles on the OCPs. Two articles on the Carbamates (Carbaryl and Mancozeb) showed consistently an inverse association between exposure and thyroid cancer. Increased risk of thyroid cancer due to the exposure to the Malathion was reported in one article on the OPs. Due to the limited current knowledge about the effect of pesticides on thyroid cancer in humans, human health policies must be implemented to control individual’s exposure to chemicals through using of botanical pesticides in agricultural. Also, more studies must be done to fill this gap of knowledge.

## Introduction

Thyroid cancer is considered as one of the most common cancers in the world as an endocrine malignancy. It is more common in women, and according to global statistics in 2018, its incidence was 10.2 per 100,000 in women and 3.1 per 100,000 in men. Over the past few decades, the thyroid cancer incidence has increased in the world, with its annual incidence tripling in the last 40 years. Approximately, 85% of all new cases of thyroid cancer are related to papillary thyroid carcinoma (PTC) [1–3]. Average annual increase in mortality rate in the United States during 1994–2013 was reported 1.1% overall and 2.9% for patients diagnosed with advanced-stage papillary thyroid cancer [4].

There is the limited knowledge about the etiology of thyroid cancer. Exposure to the ionizing radiation has been addressed as the only recognized pathogenic factor in thyroid carcinogenesis [5]. Also, the increasing incidence of differentiated thyroid cancer (DTC) can be associated to the increased production and environmental dissemination of endocrine disrupter compounds (EDCs) such as OCPs, OPs, bisphenols and phthalates [6, 7]. The obesity-related cancer burden in 2016 represented up to 9% of the cancer burden among women in North America, Europe, and the Middle East [8]. It was suggested that bisphenol A (BPA) exposure is considered as a risk factor for thyroid cancer in overweight/obese subjects [9]. Despite the widely unknown causal factors, there is a deep knowledge of underline genetics of thyroid cancer [10, 11]. Exposure to the pesticides, in the class of persistent organic pollutants (POPs), plays a role as a potentially important risk factor by affecting thyroid morphology and thyroid hormone homeostasis [1, 3]. OCPs, OPs, Carbamates and Pyrethroid are considered as the types of chemical pesticides [12]. OCPs included Dichlorodiphenyltrichloroethane (DDT) and Hexachlorobenzene (HCB) are known to disrupt thyroid hormone. After World War II and until the 1960s and 1970s, they were widely used in the farms and residential environments, and then were restricted or banned in the United States and other countries because of toxicity, carcinogenicity, and persistency. Although they are still used in many developing countries [3, 13]. Organochlorine pesticides have structural similarity to the triiodothyronine (T3) and thyroxine (T4). So they can bind to the thyroid transport proteins competitively and disrupt signaling and transport of the thyroid hormone. This disorder can reduce the thyroid hormones circulation and affect the thyroid gland via abnormal proliferation, resulted in the tumorigenesis [3, 14–16].

OPs are among the most active and widely used insecticides, currently accounting for approximately 35% of the pesticides used. The International Agency for Research on Cancer (IARC) classified Diazinon and Malathion in the class of 2A and parathion, Dichlorvos, and Tetrachlorvinphos in 2B [17]. Worldwide, about 1.8 billion people are engaged in agriculture and use approximately 1500 chemicals as pesticides. Pesticides owing to their chemical nature can cause serious environmental and health problems [18]. Global consumption of pesticides has increased to 3.5 million tons by 2020 [19]. Due to the high consumption of pesticides and their carcinogenicity, many studies have investigated the association between pesticide use and different types of cancer [1–3].

In this current study, the aim of this systematic review was to evaluate the studies that reported the epidemiological evidences for human exposure to pesticides and thyroid cancer across the world. To the best of our knowledge, this is the first systematic review to specifically address exposure to pesticides and thyroid cancer.

## Method

### Literature sources and search strategy

This systematic review was done on May 22, 2021, to review articles on the exposure to pesticides and thyroid cancer using following terms: (thyroid) AND (cancer) AND (pesticide). The period of articles published was from 2000 to 2021. A comprehensive search was conducted in electronic information resources included Web of Science, Scopus, and PubMed. Finally, most proper studies were selected according to the inclusion and exclusion criteria described as follows.

### Inclusion and exclusion criteria

For the current systematic review, we only included articles that had the following inclusion criteria:

- Original articles which were published in English in peer-reviewed journals;
- Their full text was available;
- Inspected the human exposure to pesticides and thyroid cancer via all the types of assessments included questionnaires, job-exposure matrices and bio-monitoring.

Moreover, exclusion criteria in the current review as followed:

- Inaccessible full text;
- Books;
- Presentations;
- Republished data;
- Conference papers;
- Letters to the editor;
- Reviews, systematic reviews, and meta-analysis.
- Studies focusing on thyroid diseases in general and on thyroid function alterations

### Data collection process

Articles selected according to the inclusion and exclusion criteria were firstly screened in terms of title and abstract. Then, they were further investigated independently by different two reviewers and data extracted and registered in the data collection form as followed:

- Country;
- Year of paper;
- Study design/Sample size;
- Population;
- Pesticide;
- Exposure assessment;
- Main results.

## Results

**Fig. 1:**
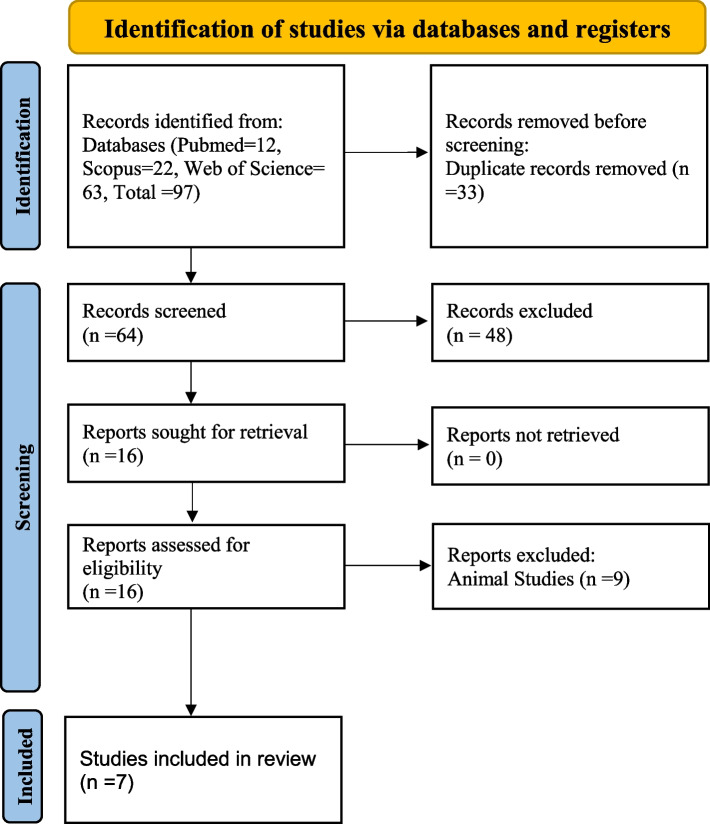
PRISMA flowchart describing the study design process

In the present systematic review, we examined the association exposure to the pesticides and thyroid cancer. Considering the search strategy that mentioned, 97 articles were totally searched through the Web of Science (*n* = 63), Scopus (*n* = 22), and PubMed (*n* = 12). Figure 1 is shown the PRISMA flow diagram of the identification of studies.

Based on the exclusion criteria, 85 articles were excluded after reading title, abstract and full-text. In the final stage, seven relevant articles were selected and included in the current systematic. The findings in this study are summarized in Table 1.

**Table 1:** Characteristics of the included studies

**No.**	**Country/Year**	**Study design/ Sample size**	**Population**	**Pesticide name (Type)**	**Exposure assessment**	**Outcome**	**Ref.**
				**Risk > 1**			
1	USA/2011	Cohort/53662	Pesticide applicators with thyroid cancer	Atrazine (OCP)^a^	Questionnaire/ Lifetime days	According to the exposed cases, there was a significant risk in the Q2^b^ (RR^c^=4.55, 1.27-16.24) and Q4^d^ (RR=4.84, 1.31-17.93) of intensity-weighted lifetime days.	[20]
2	China/2019	Case/186 Control/186	Patients with thyroid cancer	pentachlorobenzene, α-HCH^e^, HCB^f^, β-HCH, γ-HCH, δ-HCH, heptachlor, aldrin, oxy-chlordane, cis-heptachlor epoxide, trans-heptachlor epoxide, trans-chlordane,o,p′-DDE^g^, cis-chlordane, endosulfan I, trans-nonachlor, dieldrin, p,p′-DDE, o,p′-DDD^h^, endrin, endosulfan II, cis-nonachlor,p,p′-DDD, o,p′-DDT^i^, p,p′-DDT, Mirex (OCP)	Structured interview and serum sampling	Exposure to OCPs was significantly and positively associated with thyroid cancer in Chinese adults (OR^j^=1.97, 1.07−3.63).	[1]
3	Norway/2018	Case/108 Control/216	Patients with thyroid cancer	DDT metabolites (p,p′-DDE, p,p′-DDT, o,p′-DDT), Chlordane metabolite (trans-Nonachlor) (OCP)	Blood sampling	Chlordane metabolites (OR=1.78, 1.09–2.93) were positively associated with thyroid cancer.	[22]
4	USA/2015	Cohort/29325	Women pesticide applicators	Malathion (OP)^k^	Questionnaire	There was association between Malathion and increased risk of thyroid cancer (RR^l^=2.04, 1.14 -3.63).	[17]
5	USA/2021	Cohort/53096	Male pesticide applicators	Lindane and Atrazine (OCP), Carbaryl (Carbamate)	Telephone interview	Increased risk of thyroid cancer was associated with use of lindane (HR^m^ = 1.74, 1.06–2.84).	[2]
				**Risk < 1**			
6	USA/2021	Case/250 Control/250	Incident female papillary thyroid cancer (PTC) cases	OCPs: HCB, β-HCH, Oxychlordane, trans-Nonachlor, p,p’-DDE, o,p’-DDT, p,p’-DDT, Mirex	structured interview and serum sampling	OCPs were not positively associated with papillary thyroid cancer (PTC).	[3]
7	Norway/2018	Case/108 Control/216	Patients with thyroid cancer	OCPs: DDT metabolites (p,p′-DDE, p,p′-DDT, o,p′-DDT), Chlordane metabolite (trans-Nonachlor)	Blood sampling	Increasing concentration of DDT metabolites (OR=0.80, 0.66–0.98) was inversely related to the thyroid cancer.	[22]
8	Norway/2005	Cohort/523121	Patients with thyroid cancer	Mancozeb (Carbamate)	Agricultural censuses	Exposure was not associated with thyroid cancer (RR=0.87, 0.69-1.19).	[21]
9	USA/2021	Cohort/53096	Male pesticide applicators	Lindane and Atrazine (OCP), Carbaryl (Carbamate)	Telephone interview	Increased risk of thyroid cancer had an inverse association with carbaryl (HR = 0.20, 0.08–0.53).	[2]

**Note:**^a^*OCP *Organochlorine Pesticide, ^b^*Q2* Second quartile, ^c^*RR* Risk Ratio, ^d^*Q4* Fourth quartile, ^e^*HCH* Hexachlorocyclohexane, ^f^*HCB* Hexachlorobenzene, ^g^*DDE* Dichlorodiphenyldichloroethylene, ^h^*DDD* Dichlorodiphenyldichloroethane, ^i^*DDT* Dichlorodiphenyltrichloroethane, ^j^*OR* Odds Ratio, ^k^*OP* Organophosphate, ^l^Risk Ratio, ^m^*HR* Hazard Ratio

In term of the location of study, four studies were performed in the USA [2, 3, 17, 20], two studies in Norway [21, 22] and one study in China [1]. Atrazine, HCB, Hexachlorocyclohexane (HCH) isomers, Chlordane and its metabolites, DDT and its metabolites, Mirex, Pentachlorobenzene, Aldrin, Heptachlor, Heptachlor epoxides, Endosulfan I and II, Dieldrin, Endrin, and Lindane was OCPs surveyed in the included articles.

According to the results of the eligible articles reported in Table 1, some of them reported a risk more than unit as the relative risk (RR), odds ratio (OR) and hazard ratio (HR) associated to the pesticide exposure and thyroid cancer. According to the cohort study of Beane et al. on the pesticide applicators with thyroid cancer, there was a significant risk in the second percentile (RR = 4.55, 1.27–16.24) and fourth percentile (4.84, 1.31–17.93) of intensity-weighted lifetime days associated to the Atrazine, one of the most used corn herbicides [20]. In the case-control study by Han et al. in China on the relationship between exposure to OCPs and thyroid cancer via structured interview and blood sampling in Chinese adults, the results showed that exposure to OCPs was significantly and positively related to the risk of thyroid cancer (adjusted ORs for sex, age, and diabetes status = 1.97, 1.07–3.63) [1]. In the case-control study of Lerro et al. in Norway on the association between OCPs concentration included DDT metabolites (p,p′-DDE, p,p′-DDT, o,p′-DDT), Chlordane metabolite (trans-Nonachlor) in the blood samples and thyroid cancer, there was a positive association (OR = 1.78, 1.09–2.93) with thyroid cancer [21]. Personal application of specific OPs and incidence of the thyroid cancer among women pesticide applicators in the prospective Agricultural Health Study (AHS) cohort was assessed by Lerro et al. via questionnaire considering farm exposures, reproductive health history and general health. Among 30,003 applicators, 25.9% used OPs, and 718 follow-up women were identified with cancer. Malathion, the most widely used organophosphate pesticide, was related to the increased risk of cancer (RR = 2.04, 1.14–3.63) [17]. In the cohort study of Lerro et al. on the exposure to the pesticides and thyroid cancer among male applicators in USA by telephone interview, thyroid cancer risk was increased due to the application of Lindane (HR = 1.74, 1.06–2.84) [2].

Several articles included in the current study (see Table 1) showed a risks (RR, OR and HR) less than unit of the thyroid cancer after pesticide exposure. In the case-control study by Deziel et al. on the adults in the USA, which aimed to determine the association of OCPs included HCB, β-HCH, Oxychlordane, trans-Nonachlor, p,p’-DDE7, o,p’-DDT8, p,p’-DDT and Mirex with thyroid cancer through structured interview and serum sampling, OCPs were not positively associated with PTC. An inverse association between increasing concentration of DDT metabolites in the blood samples and thyroid cancer (OR = 0.80, 0.66–0.98) was reported in the case-control study of Lerro et al. in Norway [21]. Mancozeb is one of the Carbamates that most widely used as the fungicide in the Norwegian agricultural sectors. It can degrade and metabolize to the Ethylenethiourea (ETU). Published studies suggested that Mancozeb-induced ETU exposure may impair thyroid homeostasis [22]. Nordby et al. examined the relationship between exposure to Mancozeb and thyroid cancer in the patient with thyroid cancer in a cohort study with sample size 523,121 person. Mancozeb exposure was not associated with thyroid cancer (RR = 0.87, 0.69–1.19) [22]. The results of the cohort study performed by Lerro et al. showed that high use of the carbamate insecticide of Carbaryl by male pesticide applicators in USA had an inverse association with thyroid cancer (HR = 0.20, 0.08–0.53) [2].

## Discussion

Human exposure to pesticides and thyroid cancer was assessed in the present study as a systematic review of the literatures. In seven articles eligible in the systematic review, both associations of positive and negative of exposure to the pesticides and thyroid cancer were reported via different epidemiological studies. From four types of chemical pesticides namely OCPs, OPs, Pyrethroid and Carbamates, the effect of organochlorines, organophosphates and carbamates on the thyroid cancer was investigated in the included articles in the present study.

**Fig. 2:**
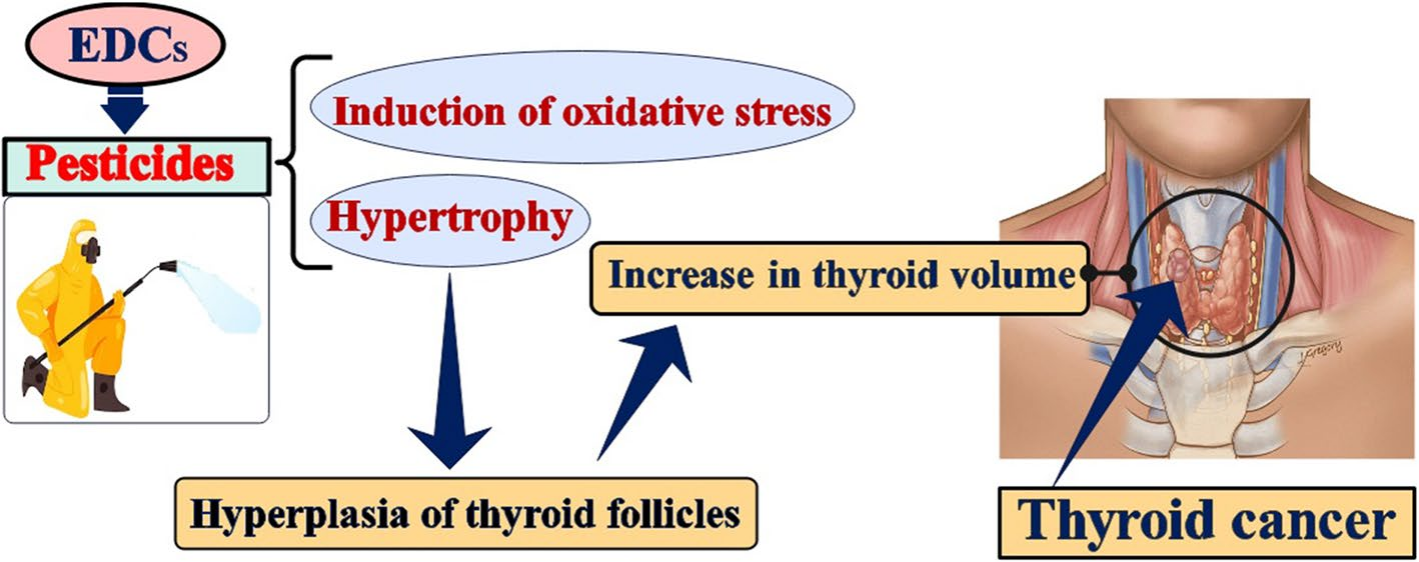
Mechanism of thyroid cancer through pesticide exposure

Some OCPs, OPs and carbamates showed a risk of thyroid cancer associated to the pesticide exposure more than unit. Several OCPs have been classified by IARC including Lindane (γ-HCCH, Group 1), DDT (Group 2A), Chlordane and HCB (Group 2B) [23]. In the case–control study by Salimi et al. on 61 patients with PTC, 70 patients with benign thyroid nodules (BTN), and 73 healthy individuals as control, it was reported that OCPs can induce thyroid tumors through induction of oxidative stress [24]. Correlation between Endosulfan exposure as a OCPs with the persistent, semi-volatile, bioaccumulative, and biomagnifying properties and thyroid cancer incidence rates (IRs) was assessed in an ecological study in the United States (US). There was a statistically significant correlation between Endosulfan use in 1992 and thyroid cancer IRs in 2012 (*r* = 0.32; *P* = 0.03) and 2014 (*r* = 0.32; *P* = 0.03) [25]. Exposure to the pesticides, may cause different cancers through various actions, including DNA damage, causing inflammation in tissues, hormonal imbalance, and turning genes on or off [26]. The thyroid hormone system has numerous sites of potential disruption by the use of multiple EDCs. The result of these interactions may decrease T4 and increase TSH that lead to hypertrophy and thus hyperplasia of thyroid follicles and thus increase in thyroid volume. In the long term, continued stimulation of follicular cells may lead to cancerous cell transformation and evolution towards follicular thyroid cancer [27]. Mechanism of thyroid cancer through pesticide exposure illustrated in Fig. 2.

Some pesticides listed in Table 1 showed a risk of thyroid cancer associated to the pesticide exposure less than unit. Despite being far from achieving statistical significance, authors reported that the number of incident thyroid cancer cases observed among both private and commercial pesticide users was higher, as compared with that expected basing on the frequencies reported for the general population. Notably, this was not observed among spouses, who are supposed to be subjected to a less degree of exposure, where the number of incident cases was even lower than expected [28, 29].

In addition to the effect of pesticides on thyroid cancer, there is some evidence on the thyroid-disruptive action of pesticides. Shrestha et al. (2019) evaluated the impacts specific pesticides on the incident hyperthyroidism in private pesticide applicators in the Agricultural Health Study. Ever use of several pesticides included organophosphate insecticide Malathion, fungicide Maneb/Mancozeb, herbicides Dicamba, Metolachlor, and Atrazine in overall sample and Chlorimuron ethyl among those ≤62 years was associated with reduced hyperthyroidism risk [30]. Shrestha et al. (2018) reported the insecticide Diazinon, the fungicides Maneb/Mancozeb, and the herbicide Metolachlor were associated with increased risk (HR ranging 1.35–2.01) and the herbicide Trifluralin with decreased risk (HR: 0.57) of hyperthyroidism in female spouses of private pesticide applicators [31].

Goldner et al. (2013) found that use of the organochlorines Chlordane, DDT, Heptachlor, Lindane, and Toxaphene, conferred higher likelihood of hypothyroidism [32]. More recently, Shrestha et al. (2018) demonstrated that the rate of hypothyroidism occurrence, as assessed as incident cases during follow-up, was significantly higher in subjects with use of the organochlorines Of Aldrin, Chlordane, Heptachlor, and Lindane, and the organophosphates Coumaphos, Diazinon, Dichlorvos, and Malathion [33].

### Future perspective to reduce the side effect of pesticides

Based on the previous studies [12], the best way to reduce the cancer and noncancerous risks of pesticides on human, it’s to reduce use pesticides in environment. Some best approaches to reduce use of pesticides in environment have been mentioned as below:

- The approach of integrated pest management (IPM) [34].
- Using of essential oils (EOs) as the green pesticides [35].
- Increasing knowledge and awareness of farmers and their families regarding reduce use of pesticides via Instruction workshops.

## Conclusion

The association between pesticide exposure and the thyroid cancer was investigated in the present review study. Inconsistent results were reported in the surveyed articles on the OCPs. Results inconsistencies are due to: a) the low number of studies; b) the different approach of exposure assessment, some of them via questionnaire, interview and blood or serum sampling; c) the different design of study; in must be noticed only prospective comparative studies assessing the differential incidence of thyroid cancer occurrence between exposed and not exposed subject can prove a causal relationship between pesticides and thyroid malignancy. So, future prospective comparative studies should address the long-term effects of pesticides in the studies among populations.

## Data Availability

Data sharing is not applicable to this article as no datasets were generated or analyzed during the current study.
